# Landscape connectivity for predicting the spread of ASF in the European wild boar population

**DOI:** 10.1038/s41598-024-53869-5

**Published:** 2024-02-10

**Authors:** Teresa Goicolea, Pablo Cisneros-Araújo, Cecilia Aguilar Vega, Jose Manuel Sánchez-Vizcaíno, MCruz Mateo-Sánchez, Jaime Bosch

**Affiliations:** 1 ETSI Montes, Forestal y del Medio Natural, Universidad Politécnica de Madrid, Madrid, Spain; 2 Department of Biology (Botany), Universidad Autónoma de Madrid, Madrid, Spain; 3 VISAVET Health Surveillance Center, Universidad Complutense de Madrid, Madrid, Spain; 4 Department of Animal HealthFaculty of Veterinary, Universidad Complutense de Madrid, Madrid, Spain

**Keywords:** African swine fever, Disease spread, Wild boar, Landscape connectivity, Animal movement, International-corridors, Probability of connectivity, Surveillance-program, Early detection, Vaccination programs, *Sus scrofa*, Ecological modelling, Ecological networks, Theoretical ecology, Ecology, Environmental sciences, Diseases, Risk factors, Disease prevention

## Abstract

African swine fever (ASF) is an infectious and highly fatal disease affecting wild and domestic swine, which is unstoppably spreading worldwide. In Europe, wild boars are one of the main drivers of spread, transmission, and maintenance of the disease. Landscape connectivity studies are the main discipline to analyze wild-species dispersal networks, and it can be an essential tool to predict dispersal-wild boar movement routes and probabilities and therefore the associated potential ASF spread through the suitable habitat. We aimed to integrate wild boar habitat connectivity predictions with their occurrence, population abundance, and ASF notifications to calculate the impact (i.e., the capacity of a landscape feature to favor ASF spread) and the risk (i.e., the likelihood of a habitat patch becoming infected) of wild boar infection across Europe. Furthermore, we tested the accuracy of the risk of infection by comparing the results with the temporal distribution of ASF cases. Our findings identified the areas with the highest impact and risk factors within Europe's central and Eastern regions where ASF is currently distributed. Additionally, the impact factor was 31 times higher on habitat patches that were infected vs non-infected, proving the utility of the proposed approach and the key role of wild boar movements in ASF-spread. All data and resulting maps are openly accessible and usable.

## Introduction

Wildlife diseases and particularly those affecting multiple hosts can greatly affect public health, the global economy, and the equilibrium of ecosystems^1^. African swine fever (ASF) is an infectious and viral disease affecting both pigs and wild boars with a very high fatality rate^2^. It has an important ecological impact on wild boar populations and devastating economic consequences for the pork industry due to its clinical outcome and trade restrictions derived from the circulation of the virus. ASF is a global threat, currently affecting all habitable continents, but most severely Europe and Asia^3^. In Europe, most ASF notifications were reported in wild boars (*Sus scrofa*), which shows the key role of wild boars in the spread, maintenance and transmission of the disease in this region^3–5^. The wide range and the high number of individuals of wild boars in Europe, as well as the lack of movement restraint make the disease more difficult and expensive to control. In Europe, ASF continues to circulate in the wild boar populations despite the considerable targeted efforts in control measures such as population control, fencing, and safe disposal of carcasses found in affected regions, and increased biosecurity measures^3,5,6,7^. Although ASF incidence has decreased for the first time in the last 8 years, surveillance is mainly passive, and, in several regions, attenuated strains are circulating^8^, so the extension of wild boar infection might be underestimated^9^. Hence, freedom from disease is yet to be foreseen and the disease is still spreading across Eurasia^5,10^. Therefore, new strategies need to be developed to improve prevention and control measures that can hamper its distribution and lead to the eradication of the disease. The absence of treatment and approved vaccines in wild boar makes it crucial to adopt alternative disease early detection and prevention measures. One important tool is to identify the areas with the worst potential consequences, with the highest risk of infection, and the routes of spread towards ASF-free regions and countries (i.e., identifying the connectivity and, ASF movement pathways or corridors for wild boar at the landscape level)^11^.

Despite the acknowledged relevance of wild boars for the maintenance and transmission of the disease, epidemiological strategies have failed to consider wild boar movements across the landscape in the pathogen spread^9^. Particularly, most studies overlooked landscape characteristics such as the availability and distribution of different land uses and covers, and species-specific traits such as dispersal capacity (e.g., maximum dispersal distance), habitat preferences (both for living and for dispersing), the occurrence of the species, and population abundance (and how is it related and linked with the previous factors described). Landscape connectivity is the scientific discipline that studies how species move across landscapes depending on the landscape traits (composition and configuration) and the species-specific characteristics^12,13^. These movements are essential for accessing resources, exchanging individuals and genes, and adapting to environmental perturbations. Therefore, a sufficient degree of movement is necessary for species' long-term survival, and landscape connectivity assessments are central to conservation science and widely utilized in biodiversity conservation initiatives^14^. However, these wildlife movements can also lead to unintended consequences such as the spread of diseases, pests, or invasive species and landscape connectivity assessments can also locate the areas that contribute the most to the spread of these threats^15–17^. Therefore, and despite being neglected in earlier studies, wild boar connectivity predictions at a large scale can be useful tools to identify important areas to control ASF spread.

This study aimed to identify spatial hotspots in terms of the impact and risk of wild boar ASF infection in Europe and locate the potential routes for international spread of the disease to other countries. We seek to produce three maps across all continental Europe pinpointing these hotspots to guide ASF control measures based on landscape connectivity analyses. To do so, we integrated habitat connectivity predictions with wild boar abundance and ASF case locations to generate three indicator maps of (1) ASF impact factor: how much the infection of each habitat patch and corridor would potentially affect the whole wild boar network; (2) ASF risk factor: the threat of infection of each habitat patch from already affected areas due to the dispersal of wild boars; and (3) international travel corridors or routes for wild boar and ASF. We additionally compared the predicted ASF risk factor to actual ASF cases at multiple periods between 2019 to 2022 to determine the accuracy of our predictions, regarding the current epidemiological situation of ASF in Europe.

## Materials and methods

### Materials

We used data from CORINE 2018^18^ at 100-m resolution to characterize the landscape and to map the different land covers and uses across Europe. We used the Copernicus Global Land Cover Map (Copernicus GLC) at 100 m^19^ for the regions of the study area where CORINE was not available (Andorra and Kaliningrad). Wild boar abundance information was extracted from a map of relative abundance across Europe categorized into four classes (from 0 = none/negligible boar abundance to 4 = high abundance) with a resolution of 1 km^20^. The data on ASF cases was obtained from ADIS and Empres-i databases from January 2019 to July 2022.

### Characterizing landscape structure for wild boars: locating habitat patches and calculating the resistance surface

For the connectivity analyses, we took into consideration the landscape structure and how wild boars perceive it. First, we defined the available habitat patches, which represent the areas with relatively homogenous environmental conditions of suitable habitat where the species could potentially reside. Given that forests are their optimal habitat^21^, we identified habitat patches as the areas covered by forests according to the land cover map (Table S1). We simplified and aggregated the habitat patches closer to 300 m, assuming that smaller distances are very easily traversed by wild boars in their daily moves^22,23^. We also discarded all patches smaller than 4.4 km^2^ to simplify the subsequent connectivity model and reduce computing times. We selected the 4.4 km^2^ threshold assuming it is the minimum area for a group of individuals to sustain their regular movements and habitation, taking into consideration the mean population home range size^23,24^. All these operations were carried out with the ArcGIS software.

The landscape surrounding the habitat patches was characterized by a resistance surface that measures the difficulty of moving through each landscape pixel^25–27^. It was achieved by associating a resistance value to each land cover class (see “Materials”), giving low resistance values to wild boar favorite land covers for moving (such as forests, natural areas, and extensive farmlands), while greater values to sub-optimal land covers (such as artificial covers or large water bodies). We determined the resistance values of each land cover class according to expert opinion and published literature^11,21^. The intensive agriculture covers were split into two classes depending on the distance to the wild boar’s preferred covers (forests and natural areas, and extensively used farmland). We associated a lower resistance value to the agricultural areas within 2 km of the preferred covers, while larger resistance values to those farther away to consider the frequent use of agroforest mosaic habitats by the species^11–39^, and assuming a mean home range of 2 km radius^26^. The specific resistance values of each class can be seen in Table S1. Later, the resistance values were projected throughout the study area according to the land cover class of each pixel, resulting in a 100 m resolution resistance surface. The resistance surface was calculated with the ArcGIS software.

### Predicting probable routes of movement

We predicted the most favorable routes of movement between habitat patches (i.e., the corridors) with the least-cost path algorithm^28,29^. This algorithm locates the corridors or paths with the least accumulated resistance between each pair of habitat patches. The least-cost path approach also measures the accumulated resistance (i.e., effective distance) through each path as an indicator of the functionality of the path (i.e., the probability of wild boars using that particular path). We delineated the corridors and calculated their effective distance with the Linkage mapper software (McRae and Kavanagh 2011), in its pair-wise mode, and setting a maximum distance of 300 km to ensure covering most long dispersal movements^23,33^. To overcome the computationally intensive calculations associated with large networks, we divided the study area in 20 regions with an overlap of 300 km between each pair of contiguous regions. We calculated the corridors for each region independently and integrated the results by merging the corridors of every region. If the corridors of the overlapping area varied depending on the considered region, we only kept those with the shortest effective distance. We also calculated the mean ratio of *effective to Euclidean* distances of all corridors (*effective distance/Euclidean distance*) as a measure of the mean resistance value found in the corridor’s cells. This ratio was used as an estimate to later transform from Euclidean to effective distances.

### Priority areas to control ASF spread

We used two criteria to identify the most critical areas for implementing ASF spread control and surveillance measures: the impact factor, and the risk factor. Both factors were calculated using connectivity metrics based on the habitat availability concept, which considers habitat patches as spaces where connectivity exists, and integrates the connectivity within and between habitat patches in a single measure^34^. Specifically, we used the probability of connectivity (PC) index, which measures the overall habitat connectivity of a landscape by estimating the probability that two randomly placed points in a landscape fall within interconnected habitat areas both through continuous habitat patches and through the connections (corridors) among them^35^.

The impact factor measured how much the infection of each habitat patch and corridor could potentially affect the whole wild boar network (set of habitat patches and corridors within Europe). To estimate this factor, . we calculated the percentage variation in the PC index (varPC) when hypothetically removing each habitat patch and corridor from the connectivity network. Therefore, higher varPC values of an habitat patch or corridor indicate greater contributions of that individual landscape element to the overall connectivity of the species^36^. Presumably, the infection of habitat patches and corridors with high connectivity contributions (i.e., high varPC), would facilitate greater disease spread. To calculate the varPC, we used the Conefor software^37^ (www.conefor.org). Conefor calculates the varPC index function of (a) the already calculated corridors’ effective distance, (b) the dispersal capacity of wild boars, and (c) relevant patches’ attributes. To parametrize the species dispersal capacity, we accounted for a maximum dispersal distance of 250 km^23,33,38^, adjusted as an effective distance with the mean *effective to Euclidean* ratio (previously estimated). We associated this maximum effective dispersal distance to a minimum probability of dispersal of 0.02. The Conefor software uses this distance-probability association to fit a negative exponential curve that provide a probability of dispersal according to the effective distance between each pair of habitat patches (the higher the corridor effective distance, the lower the probability of dispersal). To account for intrinsic attributes of habitat patches to foster ASF spread, we used the accumulated wild boar abundance of each patch (e.g., sum of the abundance^20^ of all pixels comprising the patch), assuming that more populated habitat patches would have a greater impact in ASF spread.. In this way, the larger the habitat patch and the greater abundance values within it, the larger the patch attribute and their intrinsic impact value. To manage computational intensity, we partitioned the Conefor computations across the same previous 20 subregions and integrated the results afterwards. Lastly, we min–max normalized the results on a scale from 0 to 100.

The risk factor represented the infection threat of each habitat patch from already affected habitat patches. To measure the risk factor, we used the varPC index again, but in this case, to measure the contribution of each habitat patch to the network of already infected habitat patches. Using “nodes to add” option of the Conefor software we simulated systematically adding each not-infected patch into the network of infected patches and calculated the probability of connectivity among them. We presumed that not-infected habitat patches with a greater connectivity contribution (greater varPC) were better connected to infected patches implying a higher probability or risk of infection. We designated infected habitat patches those within 2 km of a confirmed ASF observation first suspected in 2022. We only calculated the risk of infection of non-infected habitat patches within 300 km of ASF points to decrease processing times considering highly unlikely the infection of farther patches given the dispersal capacities of the species. We used the same effective distance and dispersal capacities to parametrize Conefor as in the impact factor. However, to parametrize the intrinsic attributes of habitat patches, we used the number of ASF cases within infected habitat patches, assuming that more cases increased the risk of spread. For non-infected patches, we set to 1 the attribute representing the probability of having one first case. Similar to impact factor calculations, we min–max normalized the results in a 0 to 100 scale.

To evaluate the performance of the risk factor predictions to identify potentially infected habitat patches for the following year, we compared risk-factor predictions against actual locations of ASF cases. This evaluation involved recalculating the risk factor thrice, each time considering ASF occurrences detected independently in three past periods (specifically, years 2019, 2020, and 2021). As complete data on all ASF outbreaks for the year 2023 were unavailable at the time, a direct assessment of the 2022 risk factor was not feasible. Later, we estimated the efficiency of the risk factor by comparing the risk predictions from each year with observed ASF cases of the subsequent year. To do so, we sorted habitat patches into quartiles according to their risk factor values, ranging from the first quartile corresponding to the 25% habitat patches with the lowest risk, to the 4^th^ quartile corresponding to the 25% habitat patches with the highest risk. We then determined the percentage of habitat patches that presented ASF cases in the next year within each quartile. Additionally, we compared the mean risk factor value of infected habitat patches to those of non-infected patches within a 300 km radius around ASF points.

To further identify important areas for ASF management we additionally located the potential routes of international spread of the disease to other countries by overlapping habitat patches and corridors with country borders. We also measured the current mean impact and risk factors of ASF in each country to spot countries that should soon design more restrictive strategies for preventing ASF spread by the natural spread of wild boars.

## Results

The European network of wild boar habitat was composed of 17,806 habitat patches and 34,156 corridors. The habitat patches had a very variable area (mean 96 km^2^ and standard deviation of 4146) covering a total of 1,639,019 km^2^. The corridors had a mean Euclidean distance of 5429 m (standard deviation of 11,310 m) and a mean effective distance of 39,521 m (standard deviation of 90,153 m). The effective to Euclidean ratio of corridors was 5.68 and the maximum effective dispersal distance of wild boars was 1420 cost units (k${\text{m}} \cdot resistance$) (Fig. S1). Table S1 in the supplementary material shows the resistance values used for the analyses.

**Figure 1:**
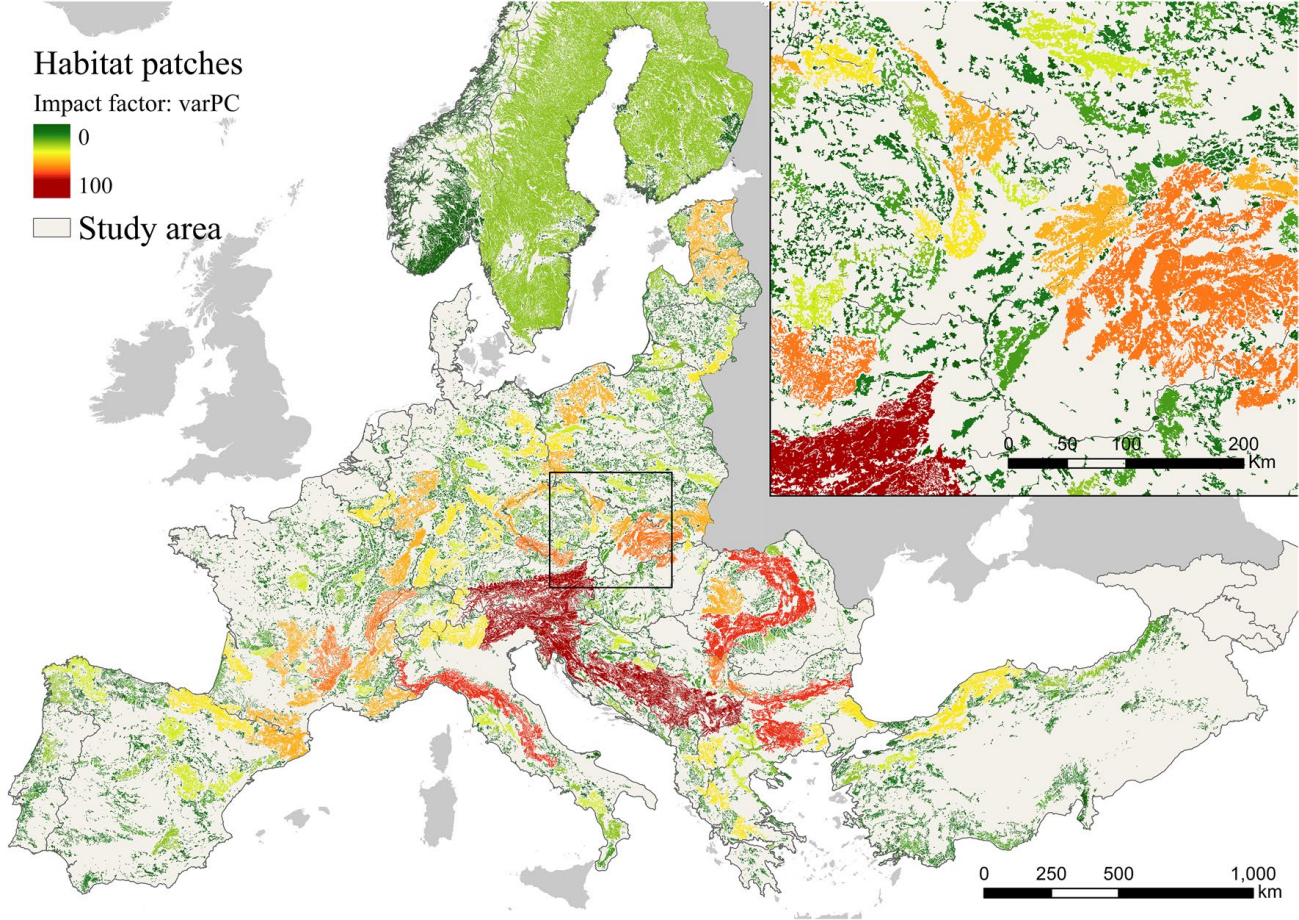
Potential impact of European habitat patches on the spread of African swine fever. Figure generated with ArcGIS Pro 2.2.0 (https://pro.arcgis.com/). A shapefile with detailed information on the potential impact factor of each habitat patch can be found in https://docs.google.com/forms/d/e/1FAIpQLSeGd8xq2L_2ZH47aVR4lryFYzKbL5rEHuv6NAZE-wrkIltxtg/viewform?usp=sf_link.

**Figure 2:**
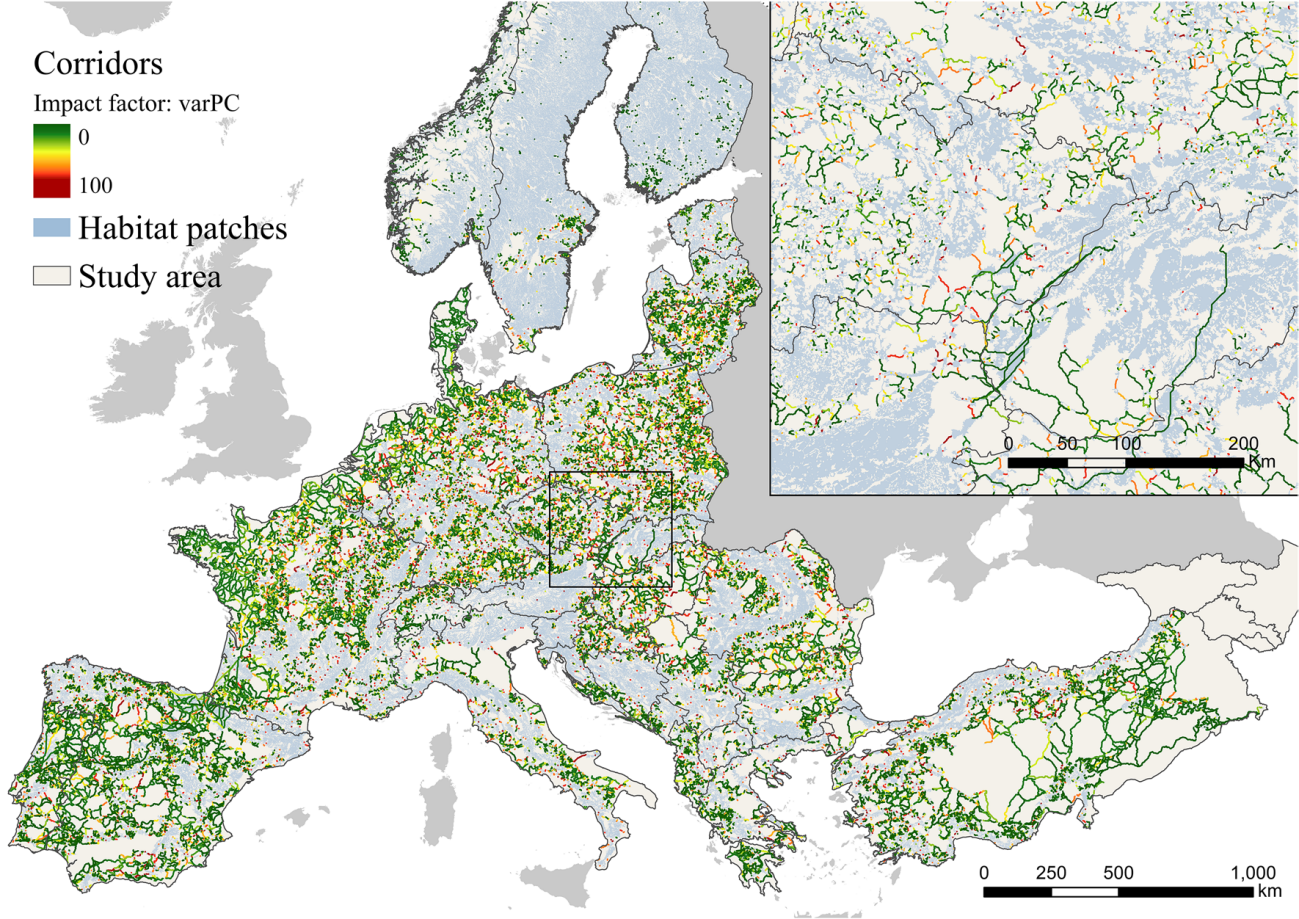
Potential impact of corridors on the spread of African swine fever in Europe. Figure generated with ArcGIS Pro 2.2.0 (https://pro.arcgis.com/). A shapefile with detailed information on the potential impact factor of each corridor can be found in https://docs.google.com/forms/d/e/1FAIpQLSeGd8xq2L_2ZH47aVR4lryFYzKbL5rEHuv6NAZE-wrkIltxtg/viewform?usp=sf_link. The risk factor of habitat patches was mainly focused on the Eastern regions (Fig. 3). Slovakia, Ukraine, and Romania, were identified as the countries with the highest mean ASF risk factor, while 13 countries had no risk factor (Table S2 in supplementary material) in the current ASF epidemiological situation in Europe. Great impact factor and great risk factor habitat patches generally did not overlap. However, some of the habitat patches and corridors with the high impact and risk factor were also international (Fig. S2 in supplementary material).

Figure 1 and 2 show the impact factor of each habitat patch and corridor respectively. The habitat patches with the highest ASF impact factor were generally distributed in central locations of Europe, while more peripheral habitat patches had a lower impact factor. Slovenia, Bosnia and Herzegovina, and Austria had the greatest mean patches impact factor, while Denmark, Netherlands, and Moldova had the lowest one (Table S2 in supplementary material) in the current ASF epidemiological situation in Europe. On the other hand, the high impact corridors were well distributed all around the study area.

**Figure 3:**
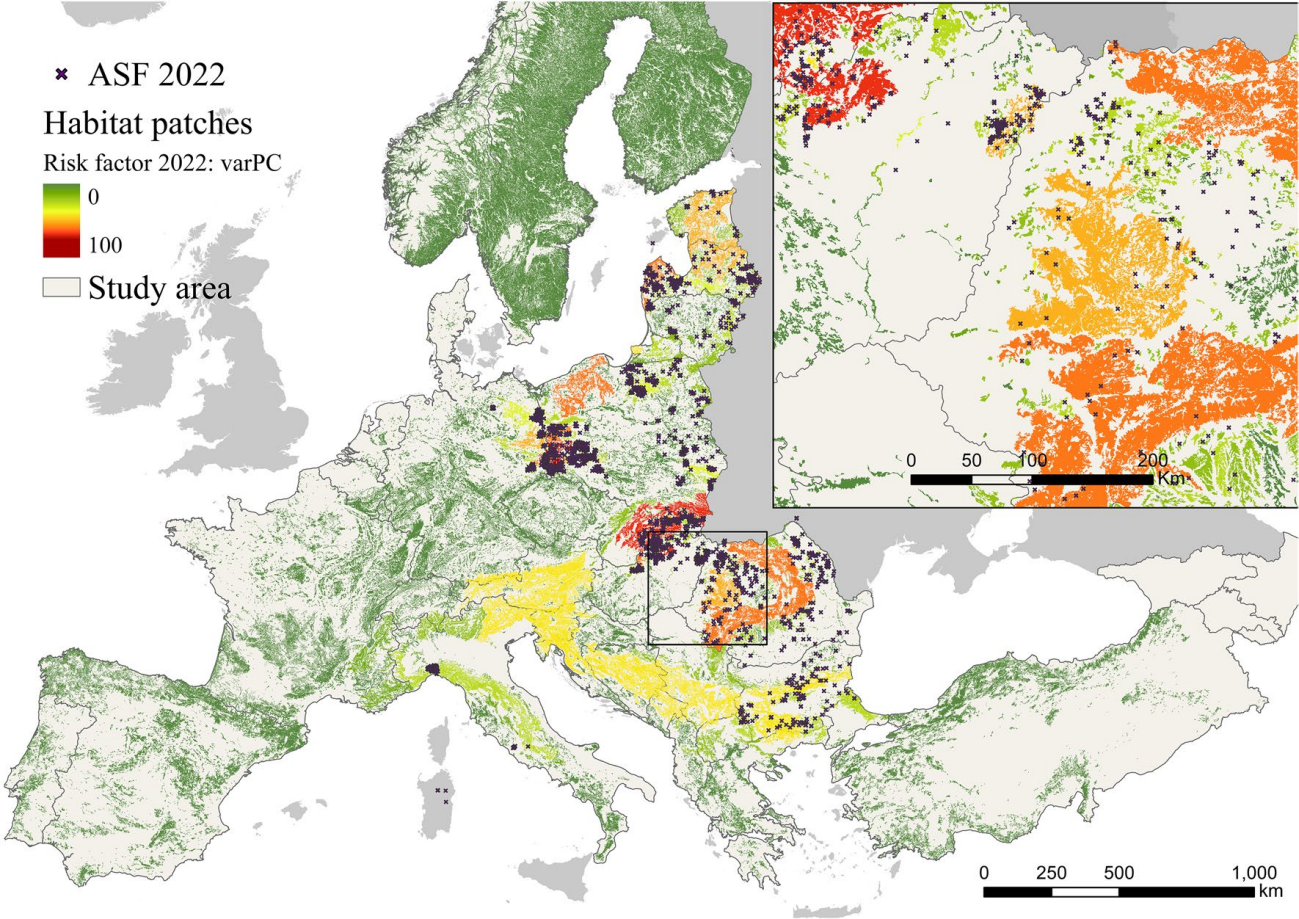
Potential risk of infection of European habitat patches from confirmed African swine fever cases detected in 2022. Figure generated with ArcGIS Pro 2.2.0 (https://pro.arcgis.com/). A shapefile with detailed information on the potential risk factor of each habitat patch can be found in https://docs.google.com/forms/d/e/1FAIpQLSeGd8xq2L_2ZH47aVR4lryFYzKbL5rEHuv6NAZE-wrkIltxtg/viewform?usp=sf_link.

For the three tested years, the risk factor was on average 31 times higher on habitat patches that actually were infected the next year compared to the patches that were not infected (Table S3). In fact, 69% (average between the three tested years) of the infected habitat patches had a very high-risk factor (they fell within the top 4th quartile), 26% had a high-risk factor (3rd quartile), and 5 and 0% had a low and very low impact factor (2nd and 1st quartiles respectively) (Table 1). In light of these results, a relationship trend has been observed between the ASF risk factor for wild boar and the spread of the disease at the landscape level in Europe.

**Table 1:** Percentage of habitat patches infected next year that fall within each quartile of the predicted risk factor for each year.

	Quartile			
	1	2	3	4
2019	0 .11	5 .50	23 .94	70 .45
2020	1 .44	7 .10	27 .68	63 .78
2021	0 .00	2 .19	25 .38	72 .43

## Discussion

This study shows that landscape connectivity studies can be a powerful tool to guide epidemiology strategies to limit the spread and persistence of diseases associated to wild population movements. This epidemiological tool, identifies and locate hotspot areas encompassing habitat patches and corridors with a potential key role in the spread of ASF in wild boar populations. Identifying these hotspots provide practical guidance, aiding to targeted surveillance, prevention, and control strategies. These efforts foster early detection and more effective disease eradication measures, potentially including future vaccination initiatives against ASF. The disease connectivity analyses are useful to anticipate the disease spread during incubation periods.

The feature that makes functional landscape connectivity studies particularly suitable to predict wildlife movements and the spread of the diseases they carry is that they explicitly consider the landscape structure and the relationship and reaction of the focal species to it^27,34^, two aspects usually neglected in wildlife disease analyses. Particularly, we considered the distribution and abundance of habitat patches, the resistance that the landscape offers to the species movements, and the dispersal capacity of the species through it. Landscape connectivity studies have mainly focused on enhancing the movements of species of special conservation concern, but this study has shown the great potential of these connectivity analyses to guide measures that limit the expansion of detrimental processes such as infectious diseases. Here, we studied the spread of ASF by wild boars, but connectivity assessments can also focus on the spread of other diseases transmitted by wild boars (such as tuberculosis classical swine fever, brucella *suis*, hepatitis E, or Aujeszky’s disease) or for any other wildlife reservoir species.

The two novel factors proposed in this study help to locate habitat patches and corridors with a potentially key role in the spread of the disease. Each of these factors provides different and critical perspectives of ASF spread that should be considered both individually and jointly.

### ASF impact factor

Given the high levels of connectivity of high-impact areas (both habitat patches and corridors) with the entire European network of wild boars, the infection of these areas could trigger a rapid spread of ASF (or other infectious disease transmitted by wild boars) to their many connected habitat patches, seriously hindering the control of the disease in Europe. The high levels of connectivity of these areas might also foster a endemic status of the disease over many years, with the risk that this entails for the rest of Europe. This information could be crucial for consideration in preventive and surveillance plans that seek to minimize the probability of introduction of ASF into wild populations as well as to reduce the spread of the disease in case such introduction should occur. Even though high-impact areas were not necessarily within imminent threat of contagious by wild boar dispersal from the known ASF outbreak, countries with a high impact factor (potential transmission hotspots) should imperiously implement additional measures to prevent the introduction of the disease in these areas both by the dispersal of wild boars from infected areas and by other anthropogenic transmission routes such as contact with ASF-contaminated food waste, domestic pigs spillover, and fomites. Furthermore, more drastic emergency actions could be designed for these high-impact areas in case of an outbreaks such as population control, fencing, ban of people entering the affected area, and correct disposal or removal of carcasses^21^. It would be advisable to minimize the wild boar—domestic pig interface and prioritize vaccination when available and approved for ASF-free but high impact areas. Considering the substantial consequences linked to ASF spread, it is important to note that preventive measures should applied across all regions of the study area, rather than solely focusing on those predicted to have a high impact factor. However, the impact factor can be a useful tool to prioritize areas for additional more strict additional measures.

We found that the countries with the highest mean impact factor were mostly located in the southeast and central parts of Europe. Furthermore, countries with a high percentage of area covered by wild boar potential habitat generally tended to have a higher impact factor, showing that the abundance of individuals and the connectivity increased with the available habitat^11^. However, larger habitat patches do not always equate to higher impact factors. Some countries such as Finland or Sweden with substantial potential habitat coverage, presented very low impact factor. This was linked to their habitat patches with low intrinsic attributes (wild boar abundance) limiting their contribution for the overall connectivity. The impact factor depends on the abundance of wild boar and the topological position and configuration of habitat patches in the connectivity network. These variables are dynamic and can rapidly shift with land use, climate, and hunting pressure changes, underscoring the importance of periodically updating the impact factor.

### ASF risk factor

Conversely, the risk factor do not depend on wild board abundance, and mainly depends on the position and configuration of the habitat patches in the landscape relative to the location of ASF cases in space and time. Habitat patches with a high-risk factor might be susceptible to immediate infection and warrant intensive and continuously monitor measures. Early detection is very important for the efficiency of eradication measures, as the longer the time lapse, the larger the infected area and the number of individuals affected^2^. Remote telemetry or biologgers that detect behavioral changes associated to ASF infection such as declines in movement, temperature or heart-rate can be excellent tools to rapidly detect the disease in high-risk areas^40^. These areas should also be subject to special strategies to prevent ASF spread, such as priority vaccination, reduced hunting pressure associated with higher movement rates^41^, or movement restriction zones especially reinforced in corridor surroundings. Careful attention should be paid to regions such as Slovakia that exhibited both high impact and risk factors, showing that its habitat are highly susceptible to infection and can rapidly spread the disease to many other interconnected habitat. Finally, the high accuracy of the risk factor showed that (i) the risk factor is a good indicator to predict where the disease is likely to spread; and (ii) wild boar movements have a key role in ASF transmission in Europe as we did not considered any other mean of ASF spread. In order to anticipate future risk factors, regular updates incorporating newly detected ASF cases are essential.

### Further improvements and future research

We presented here a broad-scale model that prioritizes regions across Europe for ASF prevention, and control plans through wild boar management, without delving into specific localized areas. Complementing this continental-scale assessment with more detailed, region-specific studies that address finer effecting wild boar dispersal and ASF spread, would provide a more nuanced prioritization of action areas. More accurate and nuanced studies demand substantial computational and information resources usually only available at localized scales, which limit the geographical extension of these analyses. Large-scale studies as this one can prioritize the crucial regions for further in-depth investigations, relying on standardized information that is not contingent on the availability of diverse data bases, or the selection of differing modeling strategies, or perspectives of analysts, managers, or politicians of smaller regions.

Additionally, only with the combination of this tool with other available management plans, the holistic approach needed to fully tackle the problematic control of the disease could be significantly improved. In fact, it must be highlighted that there are other factors that can promote the appearance and spread of ASF, for example, contaminated pork products transported by humans and consumed by wild boar can generate new notifications at great distances. In that sense, the inclusion of epidemiological models for both wild boar and domestic pigs, such as transmission dynamics^8^, wild boar—domestic pig interface^11,39^, as well as risk of exposure assessment analyses^30–32^ would be highly beneficial to improve control and prevention strategies. Other transmission routes, apart from the natural spread of the disease in wild boar studied here, should be considered for such ASF plans^42^.

The ecological models that compose the impact and risk factors try to approach and predict real patterns but are always associated to uncertainties. ASF virulent strains yield high lethality, and therefore, make highly unlikely for infected individuals with clinical signs to travel large distances. However, during the incubation period of the disease (approximately 4 days), wild boar could perform dispersive movements without symptoms and spread the disease^5^. Afterwards, the infected individual or its carcass could infect neighboring individuals and populations. If this pattern is subsequently repeated on time, the disease can spread to farther regions, leading to a slow but constant spread of the disease. Even though ASF large-distance spread is unlikely in short-medium periods by non-anthropogenic factors, it is still plausible in large periods and important to consider in ASF management strategies given the great potential consequences ensuing from ASF spread.

Studies exploring the time needed for the disease to spread different distances would offer valuable insights that complement the results presented in this study. Despite not directly assessing the time variable, the probabilities given to the corridors according to their effective distance can be a first approximation, as it associates low probabilities of dispersal to long time/distance disperse movements.

Including additional information such as more accurate dispersal rates and distances, or the behavior and landscape usage of infected individuals would provide more effective predictions. However, more refined studies would demand additional input data usually available only at localized scales and substantial computational processing times and resources. Furthermore, our validation of the risk factor predictions against the location of actual ASF cases underscores the utility of the broad scale connectivity model proposed in this study. The alignment observed between the risk factor predictions and ASF occurrences supports the efficacy of the risk factor in anticipating disease spread.

The applicability of this tool into other regions of the world, such as Asia where the disease is rapidly spreading and there is limited information on the affected wild boar populations^31^, could improve the understanding of ASF dispersion there. However, before being applied to other continents, it is important to adapt the connectivity models to address the specific relationships of the species with different landscapes. For example, in the American continent, which is widely invaded by wild pigs, the tool should be partially adapted to the ecological and behavioral differences of those pigs and could help design emergency actions against ASF if ever introduced.

## Conclusions

Movements of wild boars are likely to be one of the main factors of ASF spread in Europe. Connectivity approaches can be highly useful tools for identifying and quantifying the functionality of wild boar dispersal networks and hence, the potential spread of the disease. Based on connectivity assessments, wild boar distribution of suitable habitats, the occurrence and abundance measures, and the location of ASF cases in this wild species, we calculated two novel factors that may be very useful for controlling the disease and preventing its spread in Europe: the ASF impact factor and the ASF risk factor. First, the impact factor identified the areas whose infection would imply a wider propagation of the disease across Europe. These areas were mainly located in central regions of the continent, where additional measures should be applied to prevent the introduction of ASF through wild boar movements or anthropogenic routes. Second, the risk factor identified the areas with the highest likelihood of getting infected from actual ASF cases and were mostly located in the south-east of Europe. The habitat patches in this region are in immediate danger of infection and should be continuously monitored to control potential outbreaks of the disease. The resulting high efficacy of the risk factor to determine which areas would be infected next year proves the utility of the proposed approach and the key role of wild boar movements in ASF spread. It should be noted that the estimated risk factor is based on the actual and current situation of ASF cases in Europe and the natural movement of wild boar. However, the high spread of ASF in Europe since 2007, was also associated to other anthropogenic transmission routes such as the consumption of ASF-contaminated waste and interactions with domestic pig in backyards. Hence, the ASF risk factor should be regularly updated and especially if anthropogenic jumps are detected. This tool will help to integrate and implement more effective multidisciplinary plans for the fight and eradicate ASF improving early detection, prevention, control efforts and future potential vaccination programs in the wild boar population. Although our study focused on the case of the ASF spread in Europe, the analytical framework used in this study can be adapted and applied to other continents and diseases.

### Supplementary Information

Supplementary Information.

## Data Availability

The datasets generated during the current study are available in the Connectivity ASF WB Europe (Wildlife Ecology Diseases) repository, https://docs.google.com/forms/d/e/1FAIpQLSeGd8xq2L_2ZH47aVR4lryFYzKbL5rEHuv6NAZE-wrkIltxtg/viewform?usp=sf_link. For any data related queries, corresponding author can be contacted.
