# Supplementary material for: Landscape connectivity for predicting the spread of ASF in the European wild boar population
Source: Sci Rep. 2024 Feb 10;14:3414. doi: 10.1038/s41598-024-53869-5 (PMC10858927; doi:10.1038/s41598-024-53869-5)

**SUPPLEMENTARY MATERIAL**

*Goicolea et al., 2023. Landscape connectivity for predicting the spread of ASF in the European wild boar population*

**Fig S1.** Habitat patch area (ha), Euclidean distance (km), and Effective distance (km·resistance) of corridors. We use the logarithmic scale to represent the data. Figure generated with R 4.2.0 (https:// https://www.r-project.org/).

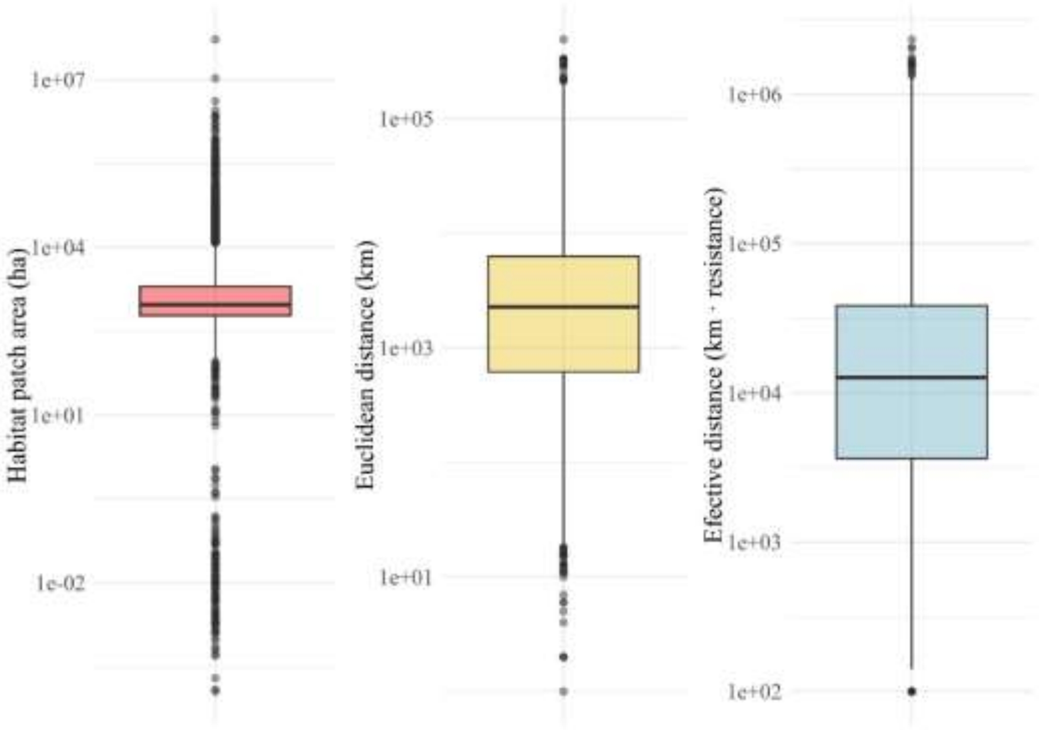


**Table S1.** Resistance values. In bold are the classes that were used as habitat patches for the connectivity analysis.

| DATA SOURCE | TYPE OF COVER | DESCRIPTION | RESISTANCE VALUE |
|---|---|---|---|
| CORINE | Artificial surfaces | Continuous urban fabric | 1000 |
| | | Discontinuous urban fabric | 100 |
| | | Industrial or commercial units | 200 |
| | | Road and rail networks and associated land | 1000 |
| | | Port areas | 1000 |
| | | Airports | 1000 |
| | | Mineral extraction sites | 1000 |
| | | Dump sites | 1000 |
| | | Construction sites | 1000 |
| | | Green urban areas | 100 |
| | | ASF spread | 200 |
| | Intensively used farmland *(> 2 km from forests and extensively used farmlands*) | Non-irrigated arable land | 100 |
| | | Permanently irrigated land | 100 |
| | | Rice fields | 100 |
| | | Vineyards | 100 |
| | | Fruit trees and berry plantations | 100 |

| | | | |
|---|---|---|---|
| | | Olive groves | 100 |
| | Intensively used farmland *(< 2 km from forests and extensively used farmlands*) | Non-irrigated arable land | 15 |
| | | Permanently irrigated land | 30 |
| | | Rice fields | 30 |
| | | Vineyards | 30 |
| | | Fruit trees and berry plantations | 30 |
| | | Olive groves | 30 |
| | Extensively used farmland | Pastures | 15 |
| | | Annual crops associated with permanent crops | 30 |
| | | Complex cultivation patterns | 15 |
| | | Land principally occupied by agriculture, with significant areas of natural vegetation | 5 |
| | Forest and natural areas | Agro-forestry areas | 5 |
| | | **Broad-leaved forest** | **1** |
| | | **Coniferous forest** | **1** |
| | | **Mixed forest** | **1** |
| | | Natural grasslands | 5 |
| | | Moors and heathland | 5 |
| | | Sclerophyllous vegetation | 1 |
| | | Transitional woodland-shrub | 1 |
| | Open spaces with little or no vegetation | Beaches, dunes, sands | 40 |
| | | Bare rocks | 40 |
| | | Sparsely vegetated areas | 40 |
| | | Burnt areas | 40 |
| | | Glaciers and perpetual snow | 1000 |
| | Wetlands | Inland marshes | 100 |
| | | Peat bogs | 100 |
| | | Salt marshes | 100 |
| | | Salines | 200 |
| | | Intertidal flats | 200 |
| | Water bodies | Water courses | 100 |
| | | Water bodies | 100 |
| | | Coastal lagoons | 200 |
| | | Estuaries | 200 |
| | | Sea and ocean | 1000 |
| Copernicus Global Land Cover Map | Artificial surfaces | Urban / built up | 1000 |
| | Farmland *(< 2 km from forests* ) | Cultivated & managed veg/agr (cropland) | 15 |
| | Open spaces | Bare / sparse vegetation | 40 |
| | Forest and natural areas | Herbaceous vegetation | 5 |
| | | Shrubs | 1 |
| | | **Closed forest, evergreen needle leaf** | **1** |
| | | **Closed forest, deciduous broad leaf** | **1** |
| | | **Closed forest, mixed** | **1** |
| | | **Closed forest, unknown** | **1** |
| | | Open forest, evergreen needle leaf | 1 |
| | | Open forest, deciduous broad leaf | 1 |
| | | Open forest, mixed | 1 |
| | | Open forest, unknown | 1 |
| | Wetlands | Herbaceous wetlands | 100 |
| | Water bodies | Permanent water bodies | 100 |

| | | |
|---|---|---|
| | Open Ocean | 1000 |

**Table S2.** Mean and standard deviation (std) of the ASF impact and risk factor and area covered by habitat per country. Countries are ordered from greater to lower combined order of impact and risk factor.

| | Impact | | Risk | | | |
|---|---|---|---|---|---|---|
| COUNTRY | MEAN | STD | MEAN | STD | Habitat area ($km^2$) | Habitat area (%) |
| Slovenia | 93.39 | 24.81 | 5.51 | 1.47 | 12434.44 | 60.12 |
| Bosnia and Herzegovina | 80.14 | 39.72 | 4.72 | 2.36 | 24101.30 | 47.48 |
| Estonia | 8.12 | 5.41 | 4.97 | 3.34 | 19669.29 | 43.28 |
| Austria | 79.33 | 39.11 | 4.60 | 2.44 | 38642.27 | 46.02 |
| Montenegro | 76.49 | 42.34 | 4.51 | 2.50 | 5710.07 | 40.67 |
| Switzerland | 7.71 | 16.33 | 0.14 | 0.90 | 11200.81 | 27.15 |
| France | 7.66 | 8.64 | 0.00 | 0.02 | 126886.09 | 23.12 |
| Serbia | 65.63 | 43.92 | 3.62 | 2.88 | 26743.49 | 30.19 |
| Greece | 6.79 | 12.02 | 0.86 | 2.17 | 23685.10 | 17.87 |
| Germany | 6.67 | 16.52 | 1.61 | 10.10 | 99988.23 | 27.99 |
| Albania | 5.81 | 20.22 | 0.26 | 1.20 | 6540.54 | 22.78 |
| Belgium | 5.26 | 3.83 | 0.00 | 0.00 | 5332.11 | 17.39 |
| Czech Republic | 5.13 | 6.48 | 0.01 | 0.03 | 24217.59 | 30.70 |
| Croatia | 46.84 | 49.57 | 2.74 | 2.94 | 17904.71 | 31.73 |
| Hungary | 4.72 | 12.50 | 7.71 | 16.49 | 15490.57 | 16.64 |
| Liechtenstein | 4.65 | 1.08 | 0.00 | 0.00 | 74.02 | 49.04 |
| Latvia | 4.35 | 5.34 | 5.72 | 5.67 | 24228.54 | 37.53 |
| Romania | 34.74 | 26.12 | 8.85 | 5.77 | 70033.95 | 29.43 |
| Italy | 31.71 | 35.58 | 1.41 | 2.05 | 70981.62 | 23.53 |
| Luxembourg | 3.96 | 4.00 | 0.00 | 0.00 | 901.57 | 34.40 |
| Poland | 3.95 | 5.61 | 8.36 | 20.38 | 89472.15 | 28.68 |
| Spain | 3.01 | 4.61 | 0.00 | 0.00 | 104739.62 | 21.02 |
| Bulgaria | 26.67 | 20.30 | 4.20 | 3.08 | 33120.13 | 29.83 |
| Slovakia | 16.87 | 9.47 | 34.71 | 19.97 | 20579.77 | 41.97 |
| Andorra | 14.50 | 0.00 | 0.00 | 0.00 | 239.42 | 50.44 |
| Ukraine | 13.46 | 19.74 | 13.27 | 11.93 | 302.24 | 0.05 |
| Macedonia | 11.40 | 25.22 | 0.55 | 1.53 | 8470.64 | 33.22 |
| Turkey | 1.77 | 2.86 | 0.01 | 0.05 | 105327.82 | 13.49 |
| Russia | 1.32 | 1.31 | 0.98 | 1.76 | 6408.33 | 0.16 |
| Belarus | 1.27 | 1.70 | 0.10 | 0.29 | 455.41 | 0.22 |
| Lithuania | 1.24 | 1.72 | 0.11 | 1.18 | 18020.12 | 27.75 |
| Sweden | 1.07 | 0.16 | 0.00 | 0.00 | 280558.14 | 62.45 |
| Finland | 1.05 | 0.24 | 0.00 | 0.00 | 224330.36 | 66.59 |
| Georgia | 0.61 | 0.23 | 0.00 | 0.00 | 1.61 | 0.00 |
| Norway | 0.39 | 0.52 | 0.00 | 0.00 | 105363.04 | 32.49 |
| Portugal | 0.19 | 0.30 | 0.00 | 0.00 | 12621.98 | 13.72 |
| Moldova | 0.04 | 0.05 | 0.00 | 0.00 | 36.56 | 0.11 |
| Netherlands | 0.03 | 0.05 | 0.00 | 0.00 | 2291.84 | 6.50 |
| Denmark | 0.00 | 0.00 | 0.00 | 0.00 | 1275.31 | 2.87 |

**Table S3.** For each year from 2019 to 2021, the ratio between the predicted mean risk factor of habitat patches that actually were infected the next year and that of patches that were not infected.

| | Mean risk factor | | |
|---|---|---|---|
| | Infected next year | Not infected next year | Ratio |
| 2019 | 1.16 | 0.08 | 15 |
| 2020 | 0.89 | 0.03 | 30 |
| 2021 | 0.96 | 0.02 | 50 |

**Fig. S2.** Habitat patches and corridors that cross several countries and contribute to the international spread of African swine fever. Figure generated with ArcGIS Pro 2.2.0 (https://pro.arcgis.com/).

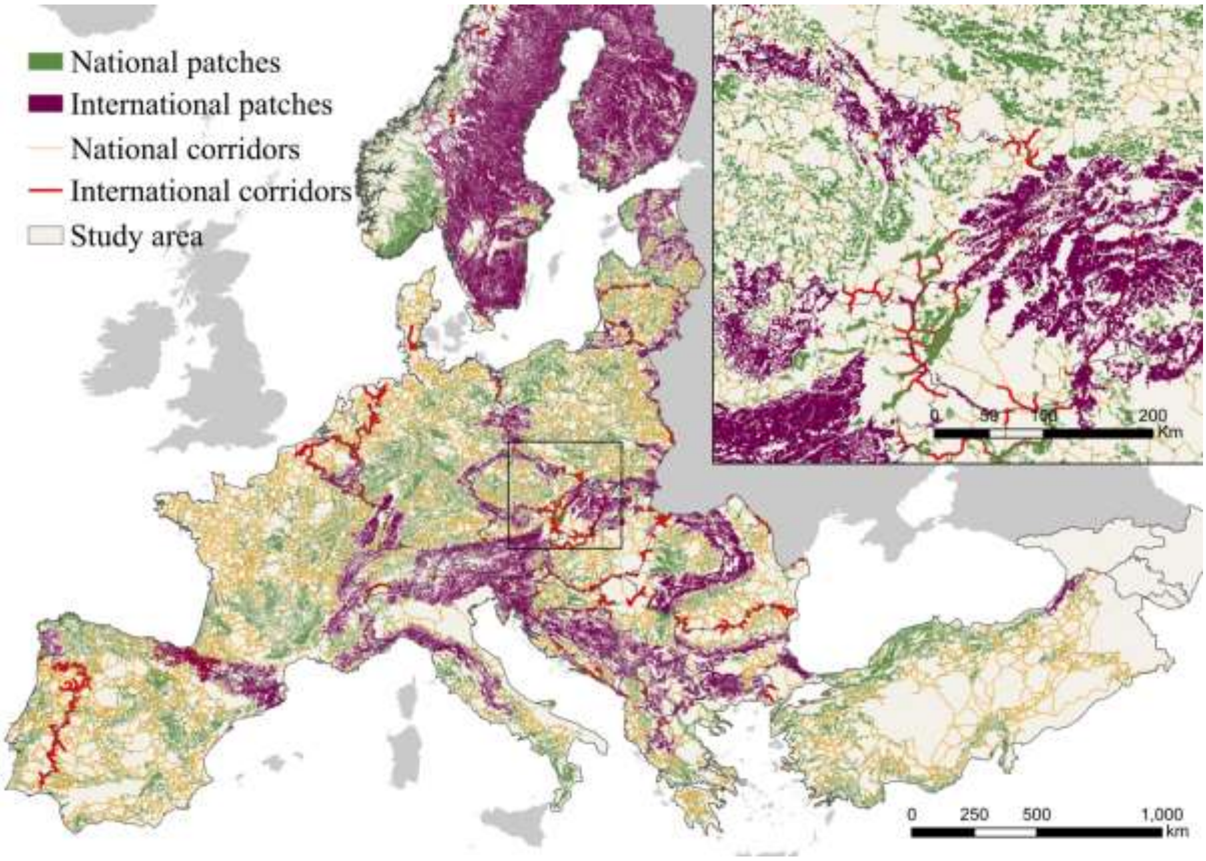

Supplement: Supplementary file 1 — Supplementary Information. [file 41598_2024_53869_MOESM1_ESM.pdf]
